# Comparing interpretable machine learning models for fall risk in middle-aged and older adults with and without pain

**DOI:** 10.1038/s41598-025-01651-6

**Published:** 2025-05-16

**Authors:** Shangmin Chen, Yongshan Gao, Lin Du, Mengzhen Min, Lei Xie, Liping Li, Xiaodong Chen, Zhigang Zhong

**Affiliations:** 1 Sports Medicine Center, The First Affiliated Hospital of Shantou University Medical College, Shantou, China; 2 School of Public Health, Shantou University, Shantou, China; 3 Sports Medicine Institute, Shantou University Medical College, Shantou, China; 4 The First Affiliated Hospital of Sun Yat sen University, Guangzhou, China; 5 Centre for Orthopaedic Research, School of Biomedical Sciences, The University of Western Australia, Nedlands, Western Australia Australia

**Keywords:** Pain, Falls, Machine learning, Risk factors, Older adults, Trauma, Geriatrics, Fracture repair, Epidemiology

## Abstract

Pain is common in middle-aged and older adults, has also been identified as a fall risk factor, whereas the mechanism of falls in pain is unclear. This study included 13,074 middle-aged and older adults from the China health and retirement longitudinal study (wave 2011–2015) to separately develop four-year fall risk prediction models for older adults with and without pain, using five machine learning algorithms with 145 input variables as candidate features. Shapley Additive exPlanations (SHAP) was used for the prediction model explanations. Adjusted logistic regression (LR) models showed that pain (OR 1.40 [1.29, 1.53]) was associated with a higher fall risk. Among pain characteristics, lower limb pain had the highest risk (OR 1.71 [1.22, 2.18]), followed by severe pain (OR 1.53 [1.36, 1.73]) and multisite pain (OR 1.43 [1.28, 1.55]). Among the fall prediction models for pain and non-pain, the LR model performed best with AUC-ROC values of 0.732 and 0.692, respectively. Common important features included fall history and height. Unique features for the pain model were functional limitation, SPPB, WBC, chronic disease score, life satisfaction, platelets, cooking fuel, and pain quantity, while marital status, age, depressive symptoms, cognitive function, hearing, rainy days, tidiness, and sleep duration were exclusive to the non-pain model. Pain characteristics are associated with falls among middle-aged and older adults. Prediction model can help identify people at high risk of falls with pain. Important features of falls differ between pain and non-pain populations, and prevention strategies should target specific populations for fall risk prediction.

**Supplementary Information:**

The online version contains supplementary material available at 10.1038/s41598-025-01651-6.

## Introduction

Falls in older people are common worldwide, and the number is increasing further^1^. Worldwide, approximately 30% of people aged 65 and older experience falls annually, and this incidence escalates with advancing age^2^. Falls are the leading cause of injury-related medical visits and death among people 65 years and above in China^3^. Apart from causing personal distress, falls present a grave healthcare issue due to their link to subsequent hospitalization, disability, and mortality^4^. Falls are widely recognized as multifactorial events, influenced by physiological decline (e.g., muscle weakness, balance impairment), chronic conditions (e.g., arthritis, diabetes), and environmental hazards (e.g., uneven surfaces, poor lighting)^1,2^. Recent studies have identified additional risk factors, including poor sleep quality and daytime sleepiness^5^, physical performance^6^, prior hospitalizations^7^, and post-acute care status^8^, further highlighting the complexity of fall mechanisms. Although many studies have shown that multifactorial interventions can effectively reduce fall risk in some older adults, there remains room for improvement, and more precise, targeted strategies are still needed^9,10^. Therefore, more efforts are needed to deepen our understanding of these risk factors and develop tailored intervention strategies for specific populations^11^.

Pain is a prevalent, debilitating, and costly condition that disproportionately affects older adults and is increasingly recognized as a critical risk factor for falls^12,13^. Notably, both pain and falls are trending younger, extending their relevance to middle-aged populations^2^. While prior studies have established an association between pain and fall risk, it remains unclear whether the underlying mechanisms differ between individuals with and without pain^13^. In addition, pain-related factors may vary considerably, such as single versus multiple pain sites, and severity of pain. Hence, building predictive models of fall risk in both pain and non-pain populations and comparing the important predictive features can facilitate insights into the mechanisms of influence and enable early and precise screening. Machine learning (ML) methods have been extensively utilized in clinical predictive models in recent years, facilitating early identification of high-risk populations^14^. Compared to traditional predictive models (e.g., Logistic regression and Cox regression), the most prominent advantage of machine learning approaches is handling high-dimensional data^15^. Moreover, the new frameworks have enhanced the interpretability of complex ML models, allowing for actionable insights from the models^16^, which could provide insights into the mechanism influencing falls among pain and non-pain populations.

This present study aims to (1) examine the association between pain and falls among middle-aged and older adults; (2) use ML methods to build 4-year fall risk prediction models among pain and non-pain, and (3) to compare the essential factors in different fall models and to develop targeted strategies for fall prevention.

## Methods

### Study design

**Fig. 1:**
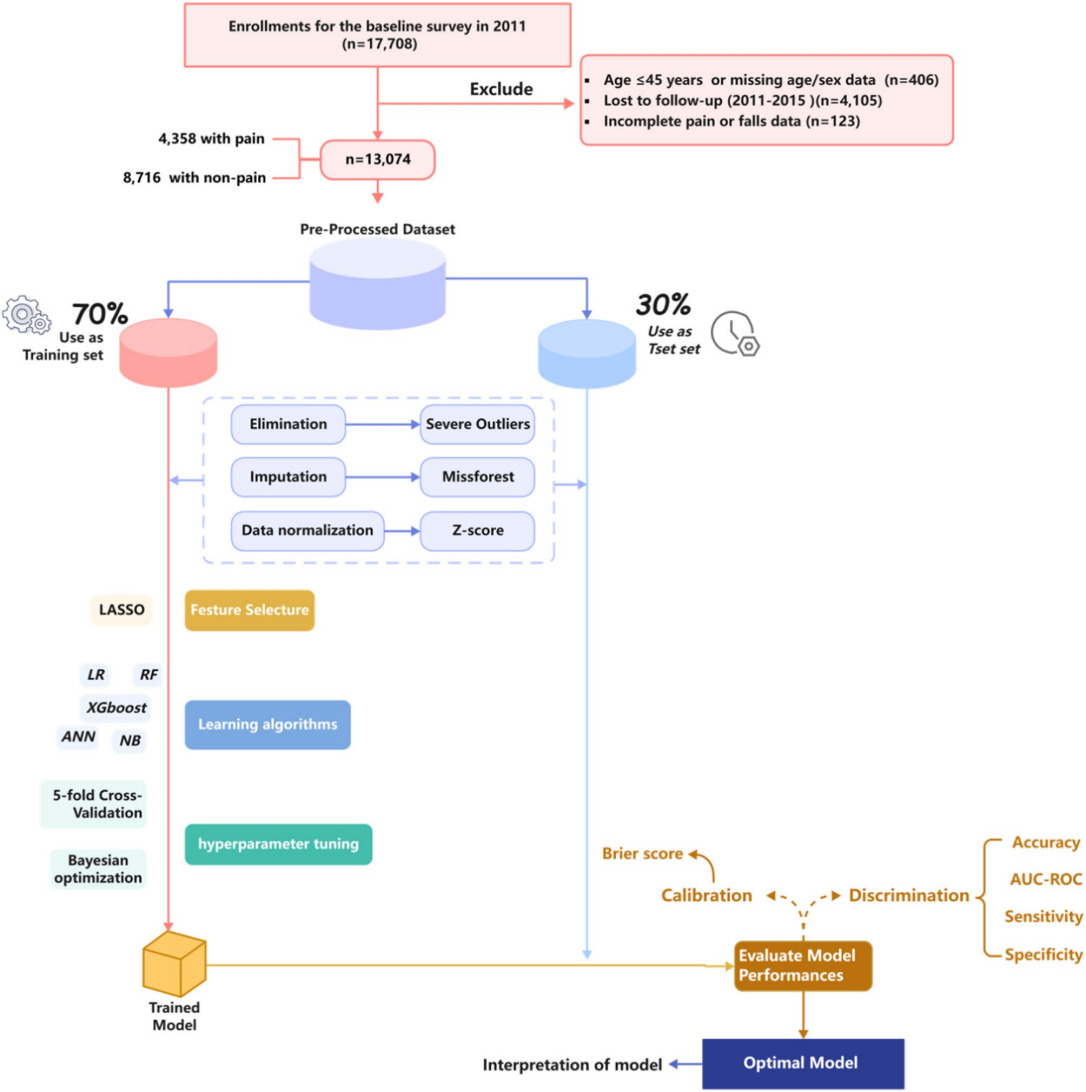
Flow chart of participant selection.

The data were obtained from the China health and retirement longitudinal study (CHARLS)^17^, publicly available at http://charls.pku.edu.cn. The CHARLS project, organized by the National Development Institute of Peking University and approved by the Ethics Review Committee, is a nationally representative longitudinal survey, launched in 2011 with the aim of monitoring the health of middle-aged and older adults (mainly those aged ≥ 45 years) in 450 villages or communities in 150 counties across 28 provinces in China^19^. The original CHARLS was approved by the Ethical Review Committee of Peking University (IRB00001052-11015), and all participants signed the informed consent at the time of participation. To ensure sample size in the prediction phase of the model, data from waves 1 to 3 (2011, 2013 and 2015) were selected. Participants were excluded if any of the following criteria were met: (1) age < 45 years and incomplete information about age and sex, (2) did not participate in the 2013 and 2015 follow-ups, (3) incomplete information about pain in wave 2011 and incomplete information about falls in wave 2013 and 2015. The final sample size of this study was 13,074 (Fig. 1).

### Outcome variables and input variables

#### Falls

A fall is an unexpected event in which an individual comes to rest on the ground, floor, or lower level^18^. Fall-related injury is an injury resulting in medical attention including hospitalization for a fall such as fractures, joint dislocation, head injury, sprain or strain, bruising, swelling, laceration, or other serious injury following a fall. The outcome of this study is based on the question from two follow-up surveys in 2013 and 2015: Have you fallen down since the last interview? and Have you fallen down seriously enough to need medical treatment?

#### Pain

Pain characteristics were assessed by three questions in wave 2011: “Are you often troubled with anybody pains?” Participants who answered “yes” were considered to have pain and were subsequently asked two follow-up questions “On what part of your body do you feel pain [head, shoulder, arm, wrist, fingers, chest, stomach, back, waist, buttocks, leg, knees, ankles, toes, and neck]?” and “How bad is your pain [mild, moderate or severe]?” Participants who reported pain in at least two or more of the 15 listed body parts were categorized as having multisite pain.

#### Input variables

Based on the literature reviews of fall risk factors and data availability in the current database, variables with more than 20% missing information were excluded from the analysis^2,11,19,20^. Notably, samples were not excluded. Finally, a total of 145 input variables were selected in the baseline (wave 2011). We divided all input variables into four sets based on the Social-Ecological Model: a framework for prevention^21^, including individual, relationship, community, and societal factors. For individual level, we included: (1) Sociodemographic variables such as age and sex; (2) Health and lifestyle variables including comorbidity and medication use (hypertension, dyslipidemia, diabetes, stroke, and other chronic conditions), smoking, alcohol consumption, sleep duration, and leisure activities; (3) Psychological variables including depressive symptoms (evaluated by the 10-item Center for epidemiological study of depression scale [CESD-10]^22^, with scores of 10 or higher was identified as exhibiting depressive symptoms) and cognitive function^23^ (including three dimensions of orientation and attention, episodic memory, and visuo-construction, with scores ranging from 0 to 31). (4) Physical condition including lung function, grip strength, Short Physical Performance Battery (SPPB), height and weight. The SPPB was evaluated by using tests of gait speed, standing balance and repeated chair stands, with score (ranging from 0 to 12) divided into three categories: poor (0–6), fair (7–9), and good (10–12)^24^. (5) Blood indices including white blood cell (WBC) count, blood urea nitrogen, platelets, and cystatin C. For relationship level, we included marital status, living alone, occupation, and residence. For community level, we included: (1) Home environment variables such as handicapped facilities, type of toilet, cooking fuel, and tidiness; (2) Community environment variables including type of road, public facilities, socio-economic status, tidiness of the roads, and industrial pollution. For societal level, we included: income, insurance and retirement. Variables were primarily collected through questionnaires and measured parameters. Assignments of variables are presented in Appendix Table S1.

### Statistical analysis

#### Association between pain characteristics and falls stages

In examining the association between pain characteristics and falls, we conducted analyses using logistic regression (LR) models, controlling for the following confounding factors based on other references^12,25,26^: sociodemographic characteristics (i.e., age, sex, education, and residence), health and lifestyle conditions (i.e., chronic disease score, polypharmacy score, vision, hearing, smoking, alcohol consumption, and body mass index [BMI]), and physical condition (i.e., SPPB). Additional sex-stratified analyses were performed to explore the association between pain-related variables and falls within each sex group separately.

#### Machine-learning stages

Based on biomedical research guideline recommendations and model features, five commonly used ML methods (logistic regression [LR], Naive Bayesian [NB], random forest [RF], extreme gradient boosting [XGBoost] and artificial neural network [ANN]) were used to build risk prediction models for falls among older adults with pain and non-pain. Among these algorithms, LR is widely recognized as a classical algorithm in statistical methods^27^. NB is a probabilistic algorithm based on probability theory, capable of handling small-sample data and addressing multi-class classification problems^28^. RF^29^ and XGBoost^30^ are ensemble techniques that utilize decision trees based on the principles of bagging and boosting to minimize the risks of underfitting and overfitting. ANN is a fundamental neural network model, proficient in parallel processing and accelerating the handling of intricate nonlinear relationships^31^.

In this study, we meticulously developed model building and evaluation, strictly adhering to the TRIPOD process^32^. The original dataset was randomly divided into training and testing dataset in a ratio of 7:3. After separating the test set, pre-processing, feature selection, and hyperparameter tuning were first performed on the training set to avoid data leakage and result bias. Data pre-processing encompasses several steps: outlier elimination, imputation, and data normalization. Among these, the MissForest algorithm was employed for missing value imputation, which effectively addresses colinearity issues and simultaneously fills continuous and categorical variables^33^. For feature selection, the method employed was the most commonly used least absolute shrinkage and selection operator^34^. Subsequently, 5-fold cross-validation and Bayesian optimization method for hyperparameter tuning were performed in each ML model, and the test set were only used to evaluate the final performance of the classifiers. For model evaluation, this study comprehensively evaluated and screened the optimal prediction models in terms of two dimensions: discrimination and calibration. The discrimination includes four metrics, i.e., accuracy, sensitivity, specificity, and area under the receiver operating characteristic curve (AUC-ROC). The primary performance measure utilized is the AUC-ROC, wherein a more excellent value denotes an enhanced model^35^. Sensitivity was considered as an additional important metric for model assessment, particularly when comparing models with similar performance across other indicators^36^. The Brier score was selected for calibration, with a lower score indicating a better fit^37^. The Shapley Additive exPlanations (SHAP) value was used to evaluate the contribution of each predictor in prediction models^38^. We also utilized partial dependence plots to visualize the impact of individual predictors on the predicted outcome, showing how variations in specific predictors influence the model’s predictions.

For the distributional characteristics of the baseline variables, the mean (standard deviation, SD) or medians (interquartile range, IQR) were used to describe the continuous variables, and number (percentage) were applied to describe categorical variables. The t-test, Wilcoxon rank sums, and Chi-square tests were selected for analysis when comparing between-group differences in falls and non-falls. DeLong’s test was conducted to evaluate the differences in AUC-ROC.

Descriptive analysis and logistic regression analyses were conducted using R4.3.1, while data preprocessing, features selection, ML model building, and evaluation were executed using Python 3.7.6. A two-sided test was utilized, and *P* < 0.05 was considered statistically significant.

## Results

A total of 17,708 participants were enrolled in the baseline survey (wave 2011). According to the exclusion criteria, the final sample was narrowed down to 13,074. Among them, 4,358 (33.3%) were with pain and 8,716 (66.7%) were with non-pain, with the pain group having a mean age of 59.6 years (SD = 9.2 years) and the non-pain group having a mean age of 58.7 years (SD = 9.4 years). The male percentages were 38.5% (1679/4358) and 52.7% (4592/8716) in two groups (Appendix Table S2). The 4-year occurrence of falls was significantly higher in middle-aged and older adults with pain than in those with non-pain (42.9 and 29.5%, *P* < 0.001) (Appendix Table S3), similar to that of fall-related injuries (45.6 and 31.4%, *P* < 0.001) (Appendix Table S4).

The adjusted Logistic regression results show that after adjusting for confounders, middle-aged and older adults with pain had a higher risk of falls than those without pain (adj. *OR* 1.40, 95% *CI* 1.29, 1.53) (Table 1). Different pain characteristics were associated with falls, including pain site, pain severity, and pain quantity. Among these, lower limb pain was associated with the highest fall risk (adj. *OR* 1.71, 95% *CI* 1.22, 2.18), followed by severe pain (adj. *OR* 1.53, 95% *CI* 1.36, 1.73) and multisite pain (*adj*. *OR* 1.43, 95% *CI* 1.28, 1.55). In fall-related injuries (Appendix Table S5), there were significant differences except for head/neck and lower limb. In the sex-stratified analysis (Appendix Table S6), there were significant differences except for single site pain in males and mild pain severity level in females.

**Table 1:** Multivariate analysis of the association between pain characteristics and falls (*N* = 13,074).

Pain characteristics	*n*	No. of falls	Model 1^a^	Model 2^b^	Model 3^c^	Model 4^d^
			Adj. OR (95% CI)			
Pain						
No	8716	2135	1.0	1.0	1.0	1.0
Yes	4358	1603	**1.79 (1.66, 1.94)**	**1.67 (1.54, 1.81)**	**1.43 (1.31, 1.56)**	**1.40 (1.29, 1.53)**
Pain site						
No pain	8716	2135	1.0	1.0	1.0	1.0
Head/neck	771	271	**1.67 (1.43, 1.95)**	**1.59 (1.36, 1.86)**	**1.38 (1.17, 1.62)**	**1.36 (1.15, 1.59)**
Trunk	990	334	**1.57 (1.36, 1.81)**	**1.49 (1.29, 1.72)**	**1.34 (1.16, 1.55)**	**1.32 (1.14, 1.52)**
Upper limb	2308	882	**1.91 (1.73, 2.10)**	**1.76 (1.59, 1.95)**	**1.45 (1.30, 1.61)**	**1.42 (1.27, 1.58)**
Lower limb	289	116	**2.01 (1.63, 2.63)**	**1.89 (1.49, 2.41)**	**1.75 (1.37, 2.24)**	**1.71 (1.33, 2.18)**
Pain severity						
No pain	8716	2135	1.0	1.0	1.0	1.0
Mild	1078	348	**1.47 (1.28, 1.69)**	**1.40 (1.21, 1.60)**	**1.25 (1.09, 1.44)**	**1.25 (1.08, 1.43)**
Moderate	1576	574	**1.77 (1.58, 1.98)**	**1.64 (1.46, 1.85)**	**1.40 (1.24, 1.58)**	**1.39 (1.23, 1.57)**
Severe	1704	681	**2.05 (1.84, 2.29)**	**1.90 (1.70, 2.13)**	**1.59 (1.41, 1.79)**	**1.53 (1.36, 1.73)**
Pain quantity						
No pain	8716	2135	1.0	1.0	1.0	1.0
Single site pain	928	308	**1.53 (1.32, 1.77)**	**1.49 (1.29, 1.73)**	**1.40 (1.20, 1.62)**	**1.37 (1.18, 1.59)**
Multisite pain	3430	1295	**1.87 (1.72, 2.04)**	**1.73 (1.58, 1.88)**	**1.44 (1.31, 1.58)**	**1.43 (1.28, 1.55)**

*Adj. OR* adjusted odds ratio, *CI* confidence interval, *SPPB* short physical performance battery.

^a^Model 1 estimated unadjusted odds ratio from logistic regression models.

^b^Model 2 was adjusted for age, sex, education, marital status, residence.

^c^Model 3 was additionally adjusted for chronic disease score, polypharmacy score, vision, hearing, smoking, alcohol consumption, and BMI.

^d^Model 4 was additionally adjusted for SPPB.

The values in bold indicate statistically significant results (*p* < 0.05).

**Fig. 2:**
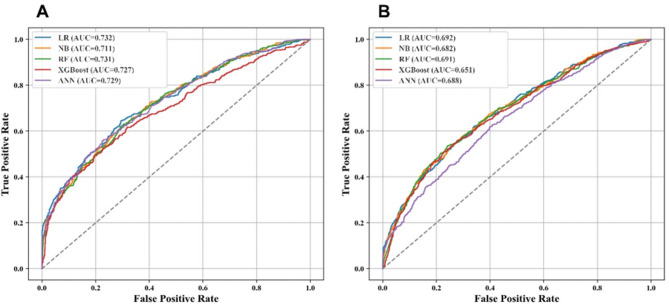
Receiver operating characteristic curve performance of five models on the test set. (**A**) Pain populations. (**B**) Non-pain populations. *ML* machine learning, *LR* logistic regression, *NB* naive Bayesian, *RF* random forest, *XGBoost* extreme gradient boosting, *ANN* artificial neural network, *AUC-ROC* area under the receiver operating characteristic curve.

Based on the association between pain and falls, our machine learning models further revealed population-specific risk profiles. During the baseline survey, 24 and 27 input variables of falls with pain and non-pain individuals were selected from the 145 candidate features through the least absolute shrinkage and selection operator regression algorithm (Appendix Table S7 and S8). The comparison of machine learning models (Table 2) indicated that LR achieved the best predictive performance, with an AUC-ROC of 0.732 in the pain population and 0.692 in the non-pain population (Fig. 2 and Appendix Table S9). Notably, the higher AUC-ROC in the pain group suggests that pain status may serve as a strong predictive factor for falls. The lowest Brier score further confirmed LR’s superior calibration (pain group: 0.197 vs. non-pain group: 0.165).

**Table 2:** Performance of the five ML models for predicting falls among pain and non-pain populations on the test set.

ML models	Threshold	AUC-ROC (95% CI)	Accuracy	Sensitivity	Specificity	Brier score
Pain						
LR	0.385	0.732 (0.695–0.766)	0.708	0.706	0.714	0.197
NB	0.239	0.711 (0.678–0.756)	0.687	0.716	0.623	0.240
RF	0.378	0.731 (0.691–0.763)	0.687	0.669	0.815	0.201
XGBoost	0.358	0.727 (0.681–0.758)	0.686	0.667	0.831	0.202
ANN	0.385	0.729 (0.689–0.763)	0.709	0.703	0.729	0.198
Non-pain						
LR	0.242	0.692 (0.647–0.738)	0.772	0.781	0.638	0.165
NB	0.223	0.682 (0.626–0.741)	0.737	0.813	0.459	0.220
RF	0.247	0.691 (0.636–0.747)	0.774	0.773	0.784	0.166
XGBoost	0.220	0.651 (0.599–0.698)	0.770	0.772	0.730	0.171
ANN	0.273	0.688 (0.638–0.743)	0.760	0.760	0.733	0.168

*ML* machine learning, *LR* logistic regression, *NB* naive Bayesian, *RF* random forest, *XGBoost* extreme gradient boosting, *ANN* artificial neural network, *AUC-ROC* area under the receiver operating characteristic curve.

**Fig. 3:**
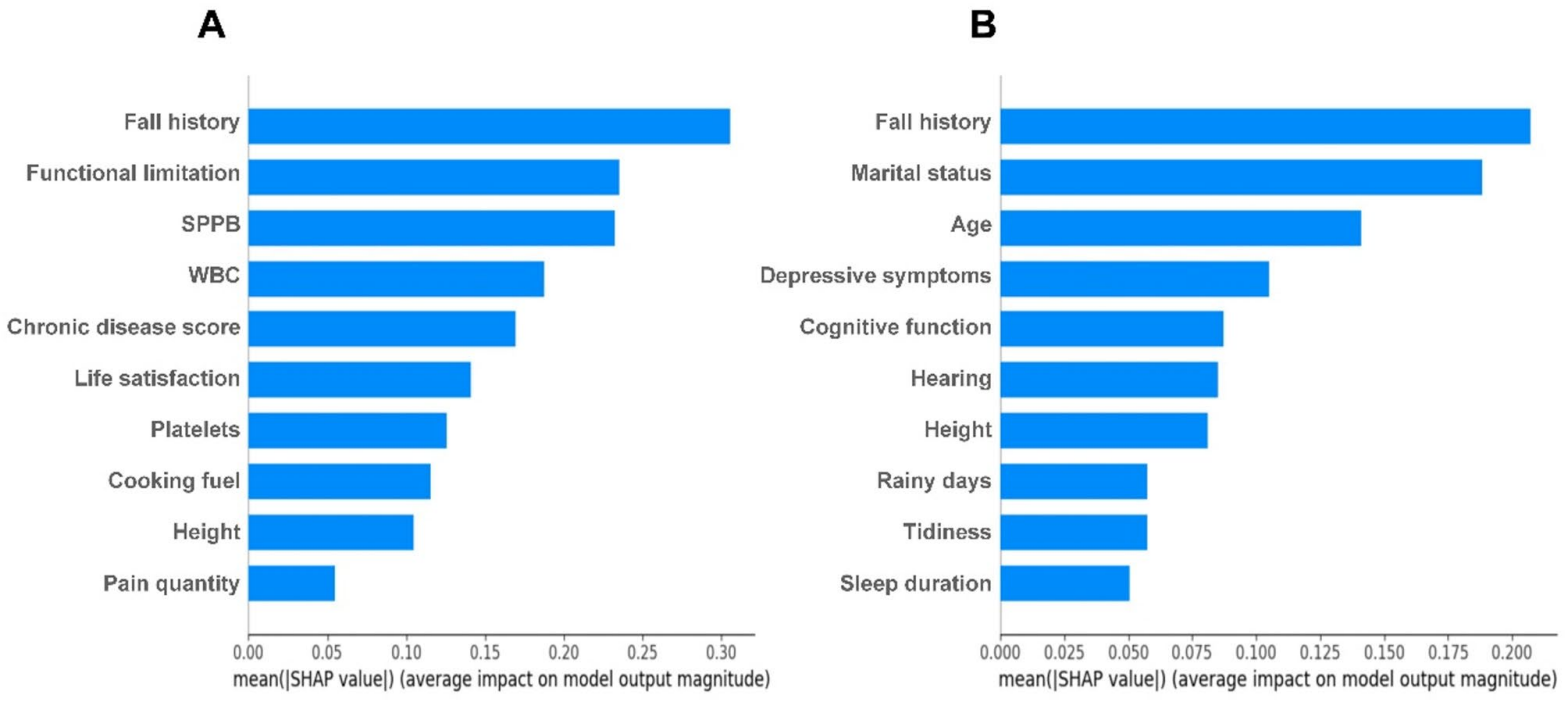
Feature Importance Ranking (top 10) with the SHAP summary plot for the logistic regression models. (**A**) Pain population model, (**B**) Non-pain population model. *SPPB* short physical performance battery, *WBC* white blood cell.

Among the top 10 important features identified by SHAP analysis (Fig. 3) based on LR model, fall history and height were shared predictors among both populations. However, the pain model uniquely prioritized biomarkers (e.g., WBC and platelets) and physical function (functional limitations and SPPB), chronic disease score, life satisfaction, cooking fuel, and pain quantity. In contrast, the non-pain model emphasized mental health factors (e.g., depressive symptoms and cognitive function), environmental factors (e.g., rainy days and tidiness), sociodemographic factors (e.g., age and marital status), physiological factors like hearing, and health behaviors like sleep duration. We further analyzed the effect of 10 important predictors in the pain population on fall prediction using Partial Dependence Plots (Appendix Figure S1). Specifically, fall history, functional limitation, decreased SPPB, elevated WBC, increased chronic disease score, lower life satisfaction, polluted cooking fuel, and increased pain quantity were associated with a higher predicted probability of falls. In contrast, improved platelets and greater height were associated with a decreased likelihood of falls. In the SHAP analysis of the pain population, 7 features consistently ranked among the top 10 predictors across five ML models (Appendix Figure S2): fall history, functional limitation, SPPB, WBC, chronic disease score, height, and pain quantity.

For the age-stratified prediction models (Appendix Table S10), the AUC-ROC of the LR model was higher in different age groups for the pain population than for the non-pain population (0.720 vs. 0.677 for 45–60 years and 0.722 vs. 0.676 for ≥ 60 years). Common predictors for both age groups in the pain population included fall history, functional limitations, chronic disease score, platelets, and WBC (Appendix Figure S3).

## Discussion

In this study of 13,074 Chinese middle-aged and older adults, we discovered significant associations between pain characteristics (i.e., status, site, severity, and quantity) and falls. In 4-year fall risk prediction models among 4,358 and 8,716 middle-aged and older adults with pain and non-pain, LR demonstrated optimal performance in both models, with an AUC-ROC of 0.73 in the pain group and 0.69 in the non-pain group. LR was chosen as the optimal model not only for its higher AUC-ROC but also for its better overall performance across metrics like sensitivity and Brier score, suggesting that its effectiveness in identifying older adults at high risk of falling. Baseline fall history and height were identified as jointly important predictive features for both groups.

The superior performance of LR over complex machine learning models (e.g., NB, RF, and XGBoost) or deep learning models (e.g., ANN) may result from its ability to balance interpretability, computational efficiency, and robustness to moderate sample sizes^27,39^, demonstrating that in clinical settings where transparency is paramount, LR provides actionable insights through feature weights, while avoiding overfitting risks associated with high-dimensional data. Besides, it’s worth noting that retrospective self-reports of falls over a two-year period may underestimate true incidence^40^, particularly for non-injurious or single falls^41^, and the four-year prediction window may not accurately reflect short-term fall risk due to changes in lifestyle, environment, and other uncontrollable factors, which can affect prediction accuracy. Therefore, caution is needed when applying this model. Future studies should prioritize prospective monitoring to improve temporal resolution and accuracy^18^.

In the SHAP analysis of LR, baseline fall history and height were identified as key predictive features shared by both groups. Additionally, the two features consistently appeared as key predictors across all five models built for the pain population. Numerous studies have shown that a history of falls is the most significant risk factor for future falls^11,42,43^. Even though a history of falls may prompt some older adults to take steps to prevent falls, for others (e.g., older adults with pain), the causes of falls are more likely to result in another fall or even fall-related injury. For the association between height and falls, study has found that older adults with height loss have an increased risk of falling^44^. On one hand, shorter older adults have a poorer field of vision^45^, while on the other hand, factors like osteoporosis, vertebral disc compression, posture issues, kyphosis, and muscle atrophy contribute to height reduction^46^. These factors collectively make older adults more prone to gait and balance issues, increasing the likelihood of falls.

In terms of physical conditions, pain can contribute to falls by affecting balance and functional activity. A systematic review of 39 articles revealed an association between pain and impaired static, dynamic, multi-component, and reactive balance among older adults^47^. Another study of 600 older adults found that multisite pain was associated with weak lower extremity function (assessed by SPPB)^48^. An Indian study found that difficulty in activities of daily living and instrumental activities of daily living with pain among older adults were 2.28 and 1.67 times higher than those without pain, respectively^49^. These indicators of physical condition are essential factors in falls and are vital in evaluating life satisfaction in older adults.

Regarding blood indices, WBC and platelets are important features for the predictive model. Most studies indicate that inflammation often accompanies the condition in pain cases, leading to an elevation in WBC levels^50^. Autoimmune disorders (e.g., platelet abnormalities) or prolonged chronic inflammation can lead to muscle weakness, decreased balance and responsiveness through increased protein metabolism, oxidative stress, and interference with neural control and endocrine regulation. Additionally, inflammation may lead to vascular damage and nerve inflammation, affecting blood flow and nerve conduction and increasing the risk of falls^51^. Some studies have also found that unclean cooking fuels are associated with health outcomes in older adults^52,53^. The underlying mechanisms driving these associations point towards inflammation and oxidative stress. Notably, pain is prevalent in chronic conditions like cancer, heart failure, kidney diseases, and musculoskeletal disorders, occasionally surpassing the prominence of their cardinal symptoms^54,55^.

In the non-pain model, the important features are mainly sociodemographic, psychological, and environmental variables. The main reason why psychological factors (i.e., depressive symptoms and cognitive function) contribute to falls is that mood problems and cognitive decline tend to lead to distraction and hesitancy to act, and a reduced ability to perceive the surrounding environment, which makes it difficult to recognize potential fall risk factors^11^. Moreover, environmental risk factors, whether in the home or community environment, are important causes of falls, especially for younger older adults^1^.

The strength of this study is that, to the authors’ knowledge, it is the first study on the association and risk prediction of pain and falls among Chinese older adults, with a large sample size and comprehensive variables (especially including community environment variables). There are also some limitations to this research. First, relying on retrospective self-reporting of falls over a two-year period may introduce recall bias. Future research should collect fall information through weekly or monthly diaries or phone follow-ups, in order to minimize the underestimation of fall incidence. Second, while our study analyzed pain by subgroups, we did not develop model predictions for different subgroups. Future studies may build predictive models for different subgroups to more accurately capture different fall risk factors and provide more precise risk predictions for subgroups. Third, while CHARLS includes a physical activity questionnaire, incomplete responses from half of the participants led us to exclude this variable. Instead, we incorporated leisure activity participation, which includes a “participation in exercise” subcomponent, and SPPB to partially compensate for this limitation. Fourth, the current ML models used in this study could identify only associations between falls and potential factors, but they could not establish causal inferences. Moreover, this study only conducted internal validation and lacked external validation to assess the model’s generalization ability. Future research should incorporate samples from various regions to externally validate the model.

In conclusion, this study suggests that older adults with pain, whether measured by pain or pain site, pain severity, or multisite pain, have a 4-year increased risk of falls. Important features of falls differ between pain and non-pain populations, and prevention strategies should target specific populations for fall risk prediction. Machine learning methods have application value in fall risk prediction in older adults with pain and can provide a scientific reference for early screening of falls, but future studies with external validation and refined datasets are needed to confirm their broader applicability.

## Electronic supplementary material

Below is the link to the electronic supplementary material.

Supplementary Material 1

## Data Availability

The datasets generated and/or analysed during the current study are available in the CHARLS repository, http://charls.pku.edu.cn.
