# Supplementary material for: Comparing interpretable machine learning models for fall risk in middle-aged and older adults with and without pain
Source: Sci Rep. 2025 May 16;15:17032. doi: 10.1038/s41598-025-01651-6 (PMC12084647; doi:10.1038/s41598-025-01651-6)
Supplement: Supplementary file 1 — Supplementary Material 1 [file 41598_2025_1651_MOESM1_ESM.docx]

**Online Supplementary Material**

**Appendix Table S1.** Assignments of Input Variables

**Appendix Table S2.** Characteristics of baseline participants and prevalence of pain (N=13,074)

**Appendix Table S3** Characteristics of Baseline Participants and the Occurrence of Falls During 4-Year Follow-Up (N=13,074)

**Appendix Table S4** Characteristics of Baseline Participants and the Occurrence of Fall-related Injuries During 4-Year Follow-Up (N=13,074)

**Appendix Table S5** Multivariate Analysis of the Association between Pain Characteristics and Fall-related Injuries (N=13,074)

**Appendix Table S6** Multivariate Analysis of the Association between Pain Characteristics and Falls Stratified by Sex

**Appendix Table S7** Univariate Analysis of Feature Selection Results of the Least Absolute Shrinkage and Selection Operator Regression among Pain Populations

**Appendix Table S8** Univariate Analysis of Feature Selection Results of the Least Absolute Shrinkage and Selection Operator Regression among Non-pain Populations

**Appendix Table S9** AUC-ROC Comparison of Different ML Models on the Test Set of Pain and Non-pain Populations

**Appendix Table S10** Performance of the LR Model for Predicting Falls among Pain Population Stratified by Age

**Appendix Figure S1** Partial Dependence Plots for Key Predictors of Fall Risk in Middle-aged and Older Adults with Pain

**Appendix Figure S2** Feature Importance Ranking (Top 10) with SHAP Summary Plots for Four Alternative Machine Learning Models among Pain Population

**Appendix Figure S3** Importance Ranking (Top 10) with the SHAP Summary Plot for the Logistic Regression Models Stratified by Age among Pain Population

**Appendix Table S1 Assignments of Input Variables**

| **Predictors** | **Evaluation** | **Assignment** |
| --- | --- | --- |
| **Individual level** |  |  |
| **Sociodemographic variables** |  |  |
| Age | Inquiry | Continuous variable |
| Sex | Inquiry | Male=1, Female=2 |
| **Health and lifestyle variables** |  |  |
| Hypertension | Inquiry | No =1, Yes =2 |
| Dyslipidemia | Inquiry | No =1, Yes =2 |
| Diabetes | Inquiry | No =1, Yes =2 |
| Cancer | Inquiry | No =1, Yes =2 |
| Chronic lung diseases | Inquiry | No =1, Yes =2 |
| Liver disease | Inquiry | No =1, Yes =2 |
| Heart diseases | Inquiry | No =1, Yes =2 |
| Stroke | Inquiry | No =1, Yes =2 |
| Kidney disease | Inquiry | No =1, Yes =2 |
| Digestive disease | Inquiry | No =1, Yes =2 |
| Psychiatric problems | Inquiry | No =1, Yes =2 |
| Memory related disease | Inquiry | No =1, Yes =2 |
| Arthritis | Inquiry | No =1, Yes =2 |
| Asthma | Inquiry | No =1, Yes =2 |
| Antihypertensive medicine | Inquiry | No =1, Yes =2 |
| Dyslipidemia medicine | Inquiry | No =1, Yes =2 |
| Diabetes medicine | Inquiry | No =1, Yes =2 |
| Cancer medicine | Inquiry | No =1, Yes =2 |
| Chronic lung medicine | Inquiry | No =1, Yes =2 |
| Liver medicine | Inquiry | No =1, Yes =2 |
| Heart medicine | Inquiry | No =1, Yes =2 |
| Stroke medicine | Inquiry | No =1, Yes =2 |
| Kidney medicine | Inquiry | No =1, Yes =2 |
| Digestive medicine | Inquiry | No =1, Yes =2 |
| Psychiatric medicine | Inquiry | No =1, Yes =2 |
| Memory-related medicine | Inquiry | No =1, Yes =2 |
| Arthritis medicine | Inquiry | No =1, Yes =2 |
| Chronic disease score | Inquiry | Continuous variable |
| Polypharmacy score | Inquiry | Continuous variable |
| Vision | Inquiry | Good=1, Fair=2, Poor=3 |
| Hearing | Inquiry | Good=1, Fair=2, Poor=3 |
| Memory | Inquiry | Good=1, Fair=2, Poor=3 |
| Health during childhood | Inquiry | Excellent=1, Very good=2, Good=3, Fair=4, Poor=5 |
| Health self-report | Inquiry | Good=1, Fair=2, Poor=3 |
| Total tooth loss | Inquiry | No=1, Yes=2 |
| Pain severity | Inquiry | Mild=1, Moderate=2, Severe=3 |
| Pain quantity | Inquiry | Continuous variable |
| Hearing disabilities | Inquiry | No =1, Yes =2 |
| Vision disabilities | Inquiry | No =1, Yes =2 |
| Brain disabilities | Inquiry | No =1, Yes =2 |
| Physical disabilities | Inquiry | No =1, Yes =2 |
| Functional limitation | Questionnaire | Continuous variable |
| BADL | Questionnaire  (BADL ≥ 1) | Normal =1, Decreased =2 |
| IADL | Questionnaire  (IAD ≥ 1) | Normal =1, Decreased =2 |
| Assistive devices | Inquiry | No =1, Yes =2 |
| Experience of falling | Inquiry | No =1, Yes =2 |
| Experience of hip fracture | Inquiry | No =1, Yes =2 |
| Experience of traffic accident | Inquiry | No =1, Yes =2 |
| Leisure activities | Inquiry | Yes =1, No =2 |
| Current smoking | Inquiry | Yes=1, No=2 |
| Current alcohol consumption | Inquiry | Yes=1, No=2 |
| Sleep duration | Inquiry | Continuous variable |
| Meals per day | Inquiry | ≥4=1, 3=2,≤2=3 |
| Weight change | Inquiry | No =1, Yes =2 |
| Life satisfaction | Inquiry | Good=1, Fair=2, Poor=3 |
| Received inpatient care | Inquiry | No =1, Yes =2 |
| **Psychological variables** |  |  |
| Depressive symptoms | Questionnaire  (CES-D-10≥10) | No =1, Yes =2 |
| Cognitive function | Questionnaire | Continuous variable |
| **Physical function variables** |  |  |
| Lung function | Measurement | Continuous variable |
| Grip strength | Measurement  (M:＜28kg, F:＜18kg) | Normal=1, Decreased=2 |
| SPPB | Measurement | Good=1, Fair=2, Poor=3 |
| Height | Measurement | Continuous variable |
| Weight | Measurement | Continuous variable |
| Hand length | Measurement | Continuous variable |
| Leg length | Measurement | Continuous variable |
| BMI | Measurement | Continuous variable |
| Abdominal obesity | Measurement  (M: ≥90cm, F: ≥85cm) | No=1, Yes=2 |
| **Blood indices variables** | 16 |  |
| White Blood Cell | Test | Continuous variable |
| Hemoglobin | Test | Continuous variable |
| Hematocrit | Test | Continuous variable |
| Mean Corpuscular Volume | Test | Continuous variable |
| Platelets | Test | Continuous variable |
| Triglycerides | Test | Continuous variable |
| Estimated Glomerular Filtration Rate | Test | Continuous variable |
| Blood Urea Nitrogen | Test | Continuous variable |
| High Density Lipoprotein Cholesterol | Test | Continuous variable |
| Low Density Lipoprotein Cholesterol | Test | Continuous variable |
| Total Cholesterol | Test | Continuous variable |
| Glucose | Test | Continuous variable |
| Uric Acid | Test | Continuous variable |
| Cystatin C | Test | Continuous variable |
| C-Reactive Protein | Test | Continuous variable |
| Glycated Hemoglobin | Test | Continuous variable |
| **Relationship level** |  |  |
| Residence | Inquiry | Urban=1, Rural=2 |
| Marital status | Inquiry | Married =1, No =2 |
| Occupation | Inquiry | Farmer=1, non-farmer=2 |
| Education | Inquiry | Illiterate=1, Primary=2, Middle and above=3 |
| Live alone | Inquiry | No =1, Yes =2 |
| **Community level** |  |  |
| **Home environment variables** |  |  |
| Area of residence |  | Continuous variable |
| Residence used for business | Inquiry | No =1, Yes =2 |
| Structure of building | Inquiry | Reinforced concrete structure=1, other=2 |
| House year | Inquiry | 0-5=1, 5-10=2, 10-20=3, 20-30=4, 30-40=5, more than 40=6 |
| Type of building | Inquiry | One story=1, multi-level=2 |
| Story | Inquiry | Continuous variable |
| Handicapped facilities | Inquiry | Yes =1, No =2 |
| Bed room | Inquiry | Yes =1, No =2 |
| Living room | Inquiry | Yes =1, No =2 |
| Toilet | Inquiry | Yes =1, No =2 |
| Kitchen | Inquiry | Yes =1, No =2 |
| Balcony | Inquiry | Yes =1, No =2 |
| Type of toilet | Inquiry | With a seat=1, Without a seat=2 |
| Flush toilets | Inquiry | Yes =1, No =2 |
| Electricity | Inquiry | Yes =1, No =2 |
| Running water | Inquiry | Yes =1, No =2 |
| Bathroom facilities | Inquiry | Yes =1, No =2 |
| Coal or natural gas | Inquiry | Yes =1, No =2 |
| Residence heating | Inquiry | Yes =1, No =2 |
| Cooking fuel | Inquiry | non-pollution=1, pollution=2 |
| Telephone | Inquiry | Yes =1, No =2 |
| Internet | Inquiry | Yes =1, No =2 |
| Tidiness | Inquiry | Clear=1, Unclear=2 |
| House temperature | Inquiry | Hot=1, Neutral =2, Cold=3 |
| **Community environment variables** |  |  |
| Main terrain | Inquiry | Plain=1, Hill=2, Mountainous region=3, plateau=4, basin=5 |
| Highest temperature | Inquiry | Continuous variable |
| Lowest temperature | Inquiry | Continuous variable |
| Rainy days in the past year | Inquiry | Continuous variable |
| Snowy days in the past year | Inquiry | Continuous variable |
| Type of road | Inquiry | Paved=1, Pathway=2, Sand-stone=3, Highway=4, other=5 |
| Roads impassable last year | Inquiry | Continuous variable |
| Bus lines are accessible | Inquiry | Continuous variable |
| Bus stop distance | Inquiry | Continuous variable |
| Train station distance | Inquiry | Continuous variable |
| Sewer system | Inquiry | Yes=1, No=2 |
| Waste disposal | Inquiry | Yes=1, No=2 |
| Type of toilet | Inquiry | Inside=1, Outside=2, Open=3 |
| Public restroom | Inquiry | Yes=1, No=2 |
| Industrial pollution | Inquiry | Poor=1, Fair=2, Good=3, Very good=4 |
| Industrial pollution nearby | Inquiry | Poor=1, Fair=2, Good=3, Very good=4 |
| Electricity supply | Inquiry | Continuous variable |
| Daily electricity supply | Inquiry | Continuous variable |
| Public facility | Inquiry | Continuous variable |
| Community facility | Inquiry | Continuous variable |
| Care center | Inquiry | Yes=1, No=2 |
| Natural disasters | Inquiry | Yes=1, No=2 |
| Open road | Inquiry | Yes=1, No=2 |
| Economic status | Inquiry | Continuous variable |
| Tidiness of the roads | Inquiry | Continuous variable |
| Construction structure | Inquiry | Continuous variable |
| Degree of crowdness | Inquiry | Continuous variable |
| Degree of handicapped access | Inquiry | Continuous variable |
| **Societal level** |  |  |
| Income | Inquiry | Yes =1, No=2 |
| Insurance | Inquiry | Yes =1, No=2 |
| Retirement | Inquiry | No =1, Yes =2 |

Note: BADL, basic activity of daily living; BMI, body mass index; IADL, instrumental activity of daily living; SPPB, Short Physical Performance Battery.

**Appendix Table S2 Characteristics of baseline participants and prevalence of pain (N=13,074)**

| **Characteristics** | **Total (n=13,074)** | **Pain**  **(n=4,358)** | **Non-pain**  **(n =8,716)** | ***P-*value** |
| --- | --- | --- | --- | --- |
| Age | 59.0 (9.3) | 59.6 (9.2) | 58.7 (9.4) | <0.001 |
| Sex |  |  |  | <0.001 |
| Male | 6,271 (48.0%) | 1,679 (38.5%) | 4,592 (52.7%) |  |
| Female | 6,803 (52.0%) | 2,679 (61.5%) | 4,124 (47.3%) |  |
| Education |  |  |  | <0.001 |
| Illiterate | 3,645 (27.9%) | 1,492 (34.2%) | 2,153 (24.7%) |  |
| Primary | 5,286 (40.4%) | 1,912 (43.9%) | 3,374 (38.7%) |  |
| Middle and above | 4,143 (31.7%) | 954 (21.9%) | 3,189 (36.6%) |  |
| Marital status |  |  |  | <0.001 |
| Married | 10,775 (82.4%) | 3,525 (80.9%) | 7,250 (83.2%) |  |
| No | 2,299 (17.6%) | 833 (19.1%) | 1,466 (16.8%) |  |
| Residence |  |  |  | <0.001 |
| Urban | 4,674 (35.8%) | 1,240 (28.5%) | 3,434 (39.4%) |  |
| Rural | 8,400 (64.2%) | 3,118 (71.5%) | 5,282 (60.6%) |  |
| Chronic disease score | 1.0 (0.0-2.0) | 2.0 (1.0- 3.0) | 1.0 (0.0-2.0) | <0.001 |
| Polypharmacy score | 0.0 (0.0-1.0) | 1.0 (0.0- 2.0) | 0.0 (0.0-1.0) | <0.001 |
| Vision |  |  |  | <0.001 |
| Good | 4,247 (32.5%) | 1,002 (23.0%) | 3,245 (37.2%) |  |
| Fair | 5,792 (44.3%) | 1,917 (44.0%) | 3,875 (44.5%) |  |
| Poor | 3,035 (23.2%) | 1,439 (33.0%) | 1,596 (18.3%) |  |
| Hearing |  |  |  | <0.001 |
| Good | 5,834 (44.6%) | 1,413 (32.4%) | 4,421 (50.7%) |  |
| Fair | 5,384 (41.2%) | 2,031 (46.6%) | 3,353 (38.5%) |  |
| Poor | 1,856 (14.2%) | 914 (21.0%) | 942 (10.8%) |  |
| BMI | 23.4 (3.5) | 23.3 (3.6) | 23.5 (3.5) | 0.051 |
| Smoking |  |  |  | <0.001 |
| Yes | 4,155 (31.8%) | 1,194 (27.4%) | 2,961 (34.0%) |  |
| No | 8,919 (68.2%) | 3,164 (72.6%) | 5,755 (66.0%) |  |
| Alcohol consumption |  |  |  | <0.001 |
| Yes | 4,378 (33.5%) | 1,242 (28.5%) | 3,136 (36.0%) |  |
| No | 8,696 (66.5%) | 3,116 (71.5%) | 5,580 (64.0%) |  |
| SPPB |  |  |  | <0.001 |
| Good | 10,283 (78.7%) | 3,118 (71.5%) | 7,165 (82.2%) |  |
| Fair | 1,953 (14.9%) | 810 (18.6%) | 1,143 (13.1%) |  |
| Poor | 838 (6.4%) | 430 (9.9%) | 408 (4.7%) |  |

Note: BMI, Body Mass Index; SPPB, Short Physical Performance Battery

**Appendix Table S3** **Characteristics of Baseline Participants and the Occurrence of Falls During 4-Year Follow-Up (N=13,074)**

| **Characteristics** | **Total (n=13,074)** | **Falls**  **(n=3,738)** | **Non-falls**  **(n =9,336)** | ***P-*value** |
| --- | --- | --- | --- | --- |
| Age | 59.0 (9.3) | 60.7 (9.7) | 58.3 (9.1) | <0.001 |
| Sex |  |  |  | <0.001 |
| Male | 6,271 (48.0%) | 1,556 (41.6%) | 4,715 (50.5%) |  |
| Female | 6,803 (52.0%) | 2,182 (58.4%) | 4,621 (49.5%) |  |
| Education |  |  |  | <0.001 |
| Illiterate | 3,645 (27.9%) | 1,236 (33.1%) | 2,409 (25.8%) |  |
| Primary | 5,286 (40.4%) | 1,554 (41.6%) | 3,732 (40.0%) |  |
| Middle and above | 4,143 (31.7%) | 948 (25.4%) | 3,195 (34.2%) |  |
| Marital status |  |  |  | <0.001 |
| Married | 10,775 (82.4%) | 2,926 (78.3%) | 7,849 (84.1%) |  |
| No | 2,299 (17.6%) | 812 (21.7%) | 1,487 (15.9%) |  |
| Residence |  |  |  | <0.001 |
| Urban | 4,674 (35.8%) | 1,239 (33.1%) | 3,435 (36.8%) |  |
| Rural | 8,400 (64.2%) | 2,499 (66.9%) | 5,901 (63.2%) |  |
| Chronic disease score | 1.0 (0.0-2.0) | 1.0 (0.0-2.0) | 1.0 (0.0-2.0) | <0.001 |
| Polypharmacy score | 0.0 (0.0-1.0) | 1.0 (0.0-1.0) | 0.0 (0.0-1.0) | <0.001 |
| Vision |  |  |  | <0.001 |
| Good | 4,247 (32.5%) | 1,062 (28.4%) | 3,185 (34.1%) |  |
| Fair | 5,792 (44.3%) | 1,670 (44.7%) | 4,122 (44.2%) |  |
| Poor | 3,035 (23.2%) | 1,006 (26.9%) | 2,029 (21.7%) |  |
| Hearing |  |  |  | <0.001 |
| Good | 5,834 (44.6%) | 1,388 (37.1%) | 4,446 (47.6%) |  |
| Fair | 5,384 (41.2%) | 1,662 (44.5%) | 3,722 (39.9%) |  |
| Poor | 1,856 (14.2%) | 688 (18.4%) | 1,168 (12.5%) |  |
| BMI | 23.4 (3.5) | 23.4 (3.7) | 23.5 (3.5) | 0.211 |
| Smoking |  |  |  | <0.001 |
| Yes | 4,155 (31.8%) | 1,068 (28.6%) | 3,087 (33.1%) |  |
| No | 8,919 (68.2%) | 2,670 (71.4%) | 6,249 (66.9%) |  |
| Alcohol consumption |  |  |  | <0.001 |
| Yes | 4,378 (33.5%) | 1,163 (31.1%) | 3,215 (34.4%) |  |
| No | 8,696 (66.5%) | 2,575 (68.9%) | 6,121 (65.6%) |  |
| SPPB |  |  |  | <0.001 |
| Good | 10,283 (78.7%) | 2,652 (70.9%) | 7,631 (81.7%) |  |
| Fair | 1,953 (14.9%) | 688 (18.4%) | 1,265 (13.5%) |  |
| Poor | 838 (6.4%) | 398 (10.6%) | 440 (4.7%) |  |
| Pain |  |  |  | <0.001 |
| Yes | 4,358 (33.3%) | 1,603 (42.9%) | 2,755 (29.5%) |  |
| No | 8,716 (66.7%) | 2,135 (57.1%) | 6,581 (70.5%) |  |
| Pain location |  |  |  | <0.001 |
| No pain | 8,716 (66.7%) | 2,135 (57.1%) | 6,581 (70.5%) |  |
| Head/neck | 771 (5.9%) | 271 (7.2%) | 500 (5.4%) | <0.001 |
| Trunk | 990 (7.6%) | 334 (8.9%) | 656 (7.0%) |  |
| Upper limb | 2,308 (17.7%) | 882 (23.6%) | 1,426 (15.3%) |  |
| Lower limb | 289 (2.2%) | 116 (3.1%) | 173 (1.9%) |  |
| Pain severity |  |  |  | <0.001 |
| No pain | 8,716 (66.7%) | 2,135 (57.1%) | 6,581 (70.5%) |  |
| Mild | 1,078 (8.2%) | 348 (9.3%) | 730 (7.8%) |  |
| Moderate | 1,576 (12.1%) | 574 (15.4%) | 1,002 (10.7%) |  |
| Severe | 1,704 (13.0%) | 681 (18.2%) | 1,023 (11.0%) |  |
| Pain quantity |  |  |  | <0.001 |
| No pain | 8,716 (66.7%) | 2,135 (57.1%) | 6,581 (70.5%) |  |
| Single site pain | 928 (7.1%) | 308 (8.2%) | 620 (6.6%) |  |
| Multisite pain | 3,430 (26.2%) | 1,295 (34.6%) | 2,135 (22.9%) |  |

Note: BMI, Body Mass Index; SPPB, Short Physical Performance Battery.

**Appendix Table S4 Characteristics of Baseline Participants and the Occurrence of Fall-related Injuries During 4-Year Follow-Up (N=13,074)**

| **Characteristics** | **Total (n=13,074)** | **Injuries**  **(n=3,738)** | **Non-Injuries**  **(n =9,336)** | ***P-*value** |
| --- | --- | --- | --- | --- |
| Age | 59.0 (9.3) | 61.1 (9.7) | 58.7 (9.2) | <0.001 |
| Sex |  |  |  | <0.001 |
| Male | 6,271 (48.0%) | 682 (39.0%) | 5,589 (49.4%) |  |
| Female | 6,803 (52.0%) | 1,067 (61.0%) | 5,736 (50.6%) |  |
| Education |  |  |  | <0.001 |
| Illiterate | 3,645 (27.9%) | 648 (37.0%) | 2,997 (26.5%) |  |
| Primary | 5,286 (40.4%) | 686 (39.2%) | 4,600 (40.6%) |  |
| Middle and above | 4,143 (31.7%) | 415 (23.7%) | 3,728 (32.9%) |  |
| Marital status |  |  |  | <0.001 |
| Married | 10,775 (82.4%) | 1,341 (76.7%) | 9,434 (83.3%) |  |
| No | 2,299 (17.6%) | 408 (23.3%) | 1,891 (16.7%) |  |
| Residence |  |  |  | <0.001 |
| Urban | 4,674 (35.8%) | 554 (31.7%) | 4,120 (36.4%) |  |
| Rural | 8,400 (64.2%) | 1,195 (68.3%) | 7,205 (63.6%) |  |
| Chronic disease score | 1.0 (0.0-2.0) | 1.0 (0.0-2.0) | 1.0 (0.0-2.0) | <0.001 |
| Polypharmacy score | 0.0 (0.0-1.0) | 1.0 (0.0-1.0) | 0.0 (0.0-1.0) | <0.001 |
| Vision |  |  |  | <0.001 |
| Good | 4,247 (32.5%) | 492 (28.1%) | 3,755 (33.2%) |  |
| Fair | 5,792 (44.3%) | 774 (44.3%) | 5,018 (44.3%) |  |
| Poor | 3,035 (23.2%) | 483 (27.6%) | 2,552 (22.5%) |  |
| Hearing |  |  |  | <0.001 |
| Good | 5,834 (44.6%) | 631 (36.1%) | 5,203 (45.9%) |  |
| Fair | 5,384 (41.2%) | 777 (44.4%) | 4,607 (40.7%) |  |
| Poor | 1,856 (14.2%) | 341 (19.5%) | 1,515 (13.4%) |  |
| BMI | 23.4 (3.5) | 23.3 (3.6) | 23.5 (3.5) | 0.013 |
| Smoking |  |  |  | <0.001 |
| Yes | 4,155 (31.8%) | 480 (27.4%) | 3,675 (32.5%) |  |
| No | 8,919 (68.2%) | 1,269 (72.6%) | 7,650 (67.5%) |  |
| Alcohol consumption |  |  |  | 0.007 |
| Yes | 4,378 (33.5%) | 536 (30.6%) | 3,842 (33.9%) |  |
| No | 8,696 (66.5%) | 1,213 (69.4%) | 7,483 (66.1%) |  |
| SPPB |  |  |  | <0.001 |
| Good | 10,283 (78.7%) | 1,222 (69.9%) | 9,061 (80.0%) |  |
| Fair | 1,953 (14.9%) | 337 (19.3%) | 1,616 (14.3%) |  |
| Poor | 838 (6.4%) | 190 (10.9%) | 648 (5.7%) |  |
| Pain |  |  |  | <0.001 |
| Yes | 4,358 (33.3%) | 797 (45.6%) | 3,561 (31.4%) |  |
| No | 8,716 (66.7%) | 952 (54.4%) | 7,764 (68.6%) |  |
| Pain location |  |  |  | <0.001 |
| No pain | 8,716 (66.7%) | 952 (54.4%) | 7,764 (68.6%) |  |
| Head/neck | 771 (5.9%) | 119 (6.8%) | 652 (5.8%) | <0.001 |
| Trunk | 990 (7.6%) | 178 (10.2%) | 812 (7.2%) |  |
| Upper limb | 2,308 (17.7%) | 452 (25.8%) | 1,856 (16.4%) |  |
| Lower limb | 289 (2.2%) | 48 (2.7%) | 241 (2.1%) |  |
| Pain severity |  |  |  | <0.001 |
| No pain | 8,716 (66.7%) | 952 (54.4%) | 7,764 (68.6%) |  |
| Mild | 1,078 (8.2%) | 164 (9.4%) | 914 (8.1%) |  |
| Moderate | 1,576 (12.1%) | 286 (16.4%) | 1,290 (11.4%) |  |
| Severe | 1,704 (13.0%) | 347 (19.8%) | 1,357 (12.0%) |  |
| Pain quantity |  |  |  | <0.001 |
| No pain | 8,716 (66.7%) | 952 (54.4%) | 7,764 (68.6%) |  |
| Single site pain | 928 (7.1%) | 145 (8.3%) | 783 (6.9%) |  |
| Multisite pain | 3,430 (26.2%) | 652 (37.3%) | 2,778 (24.5%) |  |

Note: BMI, Body Mass Index; SPPB, Short Physical Performance Battery.

**Appendix Table S5 Multivariate Analysis of the Association between Pain Characteristics and Fall-related Injuries (N=13,074)**

| Pain characteristics | n | No. of  Injuries | Model 1^a^ | Model 2^b^ | Model 3^c^ | Model 4^d^ |
| --- | --- | --- | --- | --- | --- | --- |
|  |  |  | Adj. *OR* (95% *CI*) | | | |
| Pain |  |  |  |  |  |  |
| No | 8,716 | 952 | 1.0 | 1.0 | 1.0 | 1.0 |
| Yes | 4,358 | 797 | **1.83 (1.65, 2.02)** | **1.67 (1.50, 1.85)** | **1.48 (1.32, 1.65)** | **1.46 (1.30, 1.63)** |
| Pain site |  |  |  |  |  |  |
| No pain | 8,716 | 952 | 1.0 | 1.0 | 1.0 | 1.0 |
| Head/neck | 771 | 119 | **1.49 (1.21, 1.83)** | **1.38 (1.12, 1.70)** | **1.24 (1.01, 1.53)** | 1.23 (0.99, 1.52) |
| Trunk | 990 | 178 | **1.79 (1.50, 2.13)** | **1.68 (1.41, 2.0)** | **1.55 (1.30, 1.86)** | **1.53 (1.28, 1.84)** |
| Upper limb | 2,308 | 452 | **1.99 (1.76, 2.25)** | **1.79 (1.57, 2.0)** | **1.54 (1.35, 1.77)** | **1.53 (1.33, 1.75)** |
| Lower limb | 289 | 48 | **1.62 (1.18, 2.23)** | **1.47 (1.07, 2.02)** | **1.39 (1.35, 1.77)** | 1.36 (0.99, 1.88) |
| Pain severity |  |  |  |  |  |  |
| No pain | 8,716 | 952 | 1.0 | 1.0 | 1.0 | 1.0 |
| Mild | 1078 | 164 | **1.46 (1.22, 1.75)** | **1.37 (1.14, 1.64)** | **1.26 (1.05, 1.52)** | **1.26 (1.04, 1.51)** |
| Moderate | 1576 | 286 | **1.81 (1.57, 2.09)** | **1.65 (1.43, 1.91)** | **1.47 (1.26, 1.71)** | **1.46 (1.25, 1.70)** |
| Severe | 1704 | 347 | **2.09 (1.82, 2.39)** | **1.88 (1.64, 2.16)** | **1.65 (1.42, 1.91)** | **1.61 (1.39, 1.87)** |
| Pain quantity |  |  |  |  |  |  |
| No pain | 8,716 | 952 | 1.0 | 1.0 | 1.0 | 1.0 |
| Single site pain | 928 | 145 | **1.51 (1.25, 1.83)** | **1.46 (1.21, 1.77)** | **1.39 (1.15, 1.68)** | **1.37 (1.13, 1.67)** |
| Multisite pain | 3,430 | 652 | **1.91 (1.72, 2.13)** | **1.72 (1.54, 1.93)** | **1.51 (1.33, 1.70)** | **1.49 (1.32, 1.68)** |

Note: Abbreviations: Adj. *OR* = adjusted odds ratio; C*I* = confidence interval; SPPB: Short Physical Performance Battery.

^a^ Model 1 estimated unadjusted odds ratio from logistic regression models.

^b^ Model 2 was adjusted for age, sex, education, marital status, residence.

^c^ Model 3 was additionally adjusted for chronic disease score, polypharmacy score, vision, hearing, smoking, alcohol consumption, and BMI.

^d^ Model 4 was additionally adjusted for SPPB.

The values in bold indicate statistically significant results (p < 0.05).

**Appendix Table S6 Multivariate Analysis of the Association between Pain Characteristics and Falls Stratified by Sex**

| Pain characteristics | Male Adj. *OR* (95% *CI*) | | | Female Adj. *OR* (95% *CI*) | | | |
| --- | --- | --- | --- | --- | --- | --- | --- |
|  | Model 1^a^ | | Model 2^b^ | | Model 1^a^ | Model 2^b^ | |
| Pain |  |  | |  | | |  |
| No | 1.0 | 1.0 | | 1.0 | | | 1.0 |
| Yes | **1.79 (1.58, 2.03)** | **1.43 (1.25, 1.63)** | | **1.68 (1.52, 1.86)** | | | **1.38 (1.23, 1.54)** |
| Pain site |  |  | |  | | |  |
| No pain | 1.0 | 1.0 | | 1.0 | | | 1.0 |
| Head/neck | **1.67 (1.29, 2.17)** | **1.33 (1.02, 1.74)** | | **1.56 (1.28, 1.89)** | | | **1.36 (1.11, 1.67)** |
| Trunk | **1.65 (1.34, 2.04)** | **1.37 (1.11, 1.71)** | | **1.46 (1.21, 1.77)** | | | **1.26 (1.04, 1.54)** |
| Upper limb | **1.92 (1.63, 2.25)** | **1.48 (1.24, 1.77)** | | **1.76 (1.55, 1.99)** | | | **1.37 (1.20, 1.58)** |
| Lower limb | **1.82 (1.25, 2.66)** | **1.50 (1.02, 2.20)** | | **2.16 (1.58, 2.96)** | | | **1.86 (1.35, 2.57)** |
| Pain severity |  |  | |  | | |  |
| No pain | 1.0 | 1.0 | | 1.0 | | | 1.0 |
| Mild | **1.64 (1.33, 2.03)** | **1.38 (1.11, 1.72)** | | **1.29 (1.08, 1.55)** | | | 1.16 (0.96, 1.39) |
| Moderate | **1.69 (1.40, 2.03)** | **1.37 (1.12, 1.66)** | | **1.69 (1.46, 1.96)** | | | **1.39 (1.19, 1.63)** |
| Severe | **2.01 (1.69, 2.40)** | **1.52 (1.26, 1.84)** | | **1.94 (1.69, 2.23)** | | | **1.53 (1.31, 1.78)** |
| Pain quantity |  |  | |  | | |  |
| No pain | 1.0 | 1.0 | | 1.0 | | | 1.0 |
| Single site pain | **1.39 (1.12, 1.74)** | 1.22 (0.97, 1.52) | | **1.59 (1.31, 1.93)** | | | **1.50 (1.23, 1.83)** |
| Multisite pain | **1.95 (1.70, 2.23)** | **1.52 (1.30, 1.76)** | | **1.70 (1.52, 1.90)** | | | **1.35 (1.19, 1.52)** |

Note: Abbreviations: Adj. *OR* = adjusted odds ratio; *CI* = confidence interval; SPPB: Short Physical Performance Battery.

^a^ Model 1 estimated unadjusted odds ratio from logistic regression models.

^b^ Model 2 was adjusted for age, education, marital status, residence, chronic disease score, polypharmacy score, vision, hearing, smoking, alcohol consumption, BMI and SPPB.

The values in bold indicate statistically significant results (p < 0.05).

**Appendix Table S7 Univariate Analysis of Feature Selection Results of the Least Absolute Shrinkage and Selection Operator Regression among Pain Populations**

| **Characteristics** | **Total (n=4,358)** | **Falls**  **(n=1,603)** | **Non-falls**  **(n =2,755)** | ***P-*value** |
| --- | --- | --- | --- | --- |
| **Individual level** |  |  |  |  |
| Age | 59.6 (9.2) | 60.6 (9.3) | 58.9 (9.0) | <0.001 |
| Chronic disease score | 2.0 (1.0-3.0) | 2.0 (1.0-3.0) | 2.0 (1.0-3.0) | <0.001 |
| Pain quantity | 3.0 (2.0-6.0) | 3.0 (2.0-7.0) | 3.0 (2.0-6.0) | <0.001 |
| Fall history |  |  |  | <0.001 |
| Yes | 997 (22.9%) | 547 (34.1%) | 450 (16.3%) |  |
| No | 3,361 (77.1%) | 1,056 (65.9%) | 2,305 (83.7%) |  |
| Function limitation | 14.9 (5.3) | 16.1 (5.9) | 14.3 (4.9) | <0.001 |
| Health during childhood |  |  |  | <0.001 |
| Excellent | 340 (7.8%) | 113 (7.0%) | 227 (8.2%) |  |
| Very Good | 1,527 (35.0%) | 531 (33.1%) | 996 (36.2%) |  |
| Good | 1,201 (27.6%) | 465 (29.0%) | 736 (26.7%) |  |
| Fair | 871 (20.0%) | 315 (19.7%) | 556 (20.2%) |  |
| Poor | 419 (9.6%) | 179 (11.2%) | 240 (8.7%) |  |
| Life satisfaction |  |  |  | <0.001 |
| Very good | 52 (1.2%) | 16 (1.0%) | 36 (1.3%) |  |
| Good | 620 (14.2%) | 223 (13.9%) | 397 (14.4%) |  |
| Fair | 2,732 (62.7%) | 920 (57.4%) | 1,812 (65.8%) |  |
| Poor | 769 (17.6%) | 358 (22.3%) | 411 (14.9%) |  |
| Very poor | 185 (4.2%) | 86 (5.4%) | 99 (3.6%) |  |
| SPPB |  |  |  | <0.001 |
| Good | 3,118 (71.5%) | 1,045 (65.2%) | 2,073 (75.2%) |  |
| Fair | 810 (18.6%) | 325 (20.3%) | 485 (17.6%) |  |
| Poor | 430 (9.9%) | 233 (14.5%) | 197 (7.2%) |  |
| Height | 156.4 (8.1) | 155.4 (8.0) | 157.0 (8.0) | <0.001 |
| Leg length | 47.7 (14.8) | 47.2 (3.6) | 48.0 (8.3) | 0.101 |
| White Blood Cell | 6.3 (1.7) | 6.4 (1.8) | 6.2 (1.6) | 0.010 |
| Mean Corpuscular Volume | 90.7 (7.5) | 90.8 (7.5) | 90.6 (7.6) | 0.519 |
| Platelets | 213.3 (65.8) | 211.8 (65.5) | 214.1 (65.9) | 0.256 |
| Glycated Hemoglobin | 5.2 (0.7) | 5.3 (0.7) | 5.2 (0.7) | 0.004 |
| **Community level** |  |  |  |  |
| Story | 1.0 (1.0-1.0) | 1.0 (1.0-1.0) | 1.0 (1.0-1.0) | 0.042 |
| Toilet |  |  |  | 0.738 |
| Yes | 3,037 (69.7%) | 1,122 (70.0%) | 1,915 (69.5%) |  |
| No | 1,321 (30.3%) | 481 (30.0%) | 840 (30.5%) |  |
| Cooking fuel |  |  |  | <0.001 |
| Pollution | 3,014 (69.2%) | 1,165 (72.7%) | 1,849 (67.1%) |  |
| Non-pollution | 1,344 (30.8%) | 438 (27.3%) | 906 (32.9%) |  |
| Tidiness |  |  |  | 0.009 |
| Clear | 2,716 (62.3%) | 959 (59.8%) | 1,757 (63.8%) |  |
| Unclear | 1,642(37.7) | 644 (40.2%) | 998 (36.2%) |  |
| Main terrain |  |  |  | 0.014 |
| Plain | 1,490 (34.2%) | 506 (31.6%) | 984 (35.7%) |  |
| Hill | 1,378 (31.6%) | 529 (33.0%) | 849 (30.8%) |  |
| Mountainous region | 1,112 (25.5%) | 440 (27.4%) | 672 (24.4%) |  |
| Plateau | 242 (5.6%) | 78 (4.9%) | 164 (6.0%) |  |
| Basin | 136 (3.1%) | 50 (3.1%) | 86 (3.1%) |  |
| Distance to the train station | 40.0 (16.0-80.0) | 40.0 (18.0-80.0) | 40.0 (15.0-76.0) | 0.009 |
| Industrial pollution |  |  |  | 0.001 |
| Poor | 102 (2.3%) | 28 (1.7%) | 74 (2.7%) |  |
| Fair | 474 (10.9%) | 170 (10.6%) | 304 (11.0%) |  |
| Good | 758 (17.4%) | 240 (15.0%) | 518 (18.8%) |  |
| Very good | 3,024 (69.4%) | 1,165 (72.7%) | 1,859 (67.5%) |  |
| Economic status | 4.0 (3.0-4.0) | 4.0 (2.0-4.0) | 4.0 (3.0-4.0) | 0.038 |
| Tidiness of the roads | 4.0 (3.0-5.0) | 4.0 (3.0-5.0) | 4.0 (3.0-5.0) | 0.056 |
| Degree of handicapped access | 1.0 (1.0-2.0) | 1.0 (1.0-2.0) | 1.0 (1.0-2.0) | 0.006 |

Note: SPPB, Short Physical Performance Battery.

**Appendix Table S8 Univariate Analysis of Feature Selection Results of the Least Absolute Shrinkage and Selection Operator Regression among Non-pain Populations**

| **Characteristics** | **Total (n=8,716)** | **Falls**  **(n=2,135)** | **Non-falls**  **(n =6,581)** | ***P-*value** |
| --- | --- | --- | --- | --- |
| **Individual level** |  |  |  |  |
| Age | 58.7 (9.4) | 60.7 (10.0) | 58.1 (9.1) | <0.001 |
| Chronic disease score | 1.0 (0.0-2.0) | 1.0 (0.0-2.0) | 1.0 (0.0-2.0) | <0.001 |
| Polypharmacy score | 0.0 (0.0-1.0) | 0.0 (0.0-1.0) | 0.0 (0.0-1.0) | <0.001 |
| Fall history |  |  |  | <0.001 |
| Yes | 965 (11.1%) | 440 (20.6%) | 525 (8.0%) |  |
| No | 7,751 (88.9%) | 1,695 (79.4%) | 6,056 (92.0%) |  |
| Hearing |  |  |  | <0.001 |
| Good | 4,421 (50.7%) | 944 (44.2%) | 3,477 (52.8%) |  |
| Fair | 3,353 (38.5%) | 878 (41.1%) | 2,475 (37.6%) |  |
| Poor | 942 (10.8%) | 313 (14.7%) | 629 (9.6%) |  |
| Health during childhood |  |  |  | <0.001 |
| Excellent | 882 (10.1%) | 188 (8.8%) | 694 (10.5%) |  |
| Very Good | 3,393 (38.9%) | 780 (36.5%) | 2,613 (39.7%) |  |
| Good | 2,513 (28.8%) | 622 (29.1%) | 1,891 (28.7%) |  |
| Fair | 1,438 (16.5%) | 389 (18.2%) | 1,049 (15.9%) |  |
| Poor | 490 (5.6%) | 156 (7.3%) | 334 (5.1%) |  |
| Sleep duration | 7.2 (1.9) | 6.9 (2.0) | 7.3 (1.8) | <0.001 |
| Meals per day |  |  |  | 0.003 |
| ≥4 | 149 (1.7%) | 32 (1.5%) | 117 (1.8%) |  |
| 3 | 7,623 (87.5%) | 1,829 (85.7%) | 5,794 (88.0%) |  |
| ≤2 | 944 (10.8%) | 274 (12.8%) | 670 (10.2%) |  |
| Health self-report |  |  |  | <0.001 |
| Good | 4,941 (56.7%) | 1,092 (51.1%) | 3,849 (58.5%) |  |
| Fair | 2,968 (34.1%) | 789 (37.0%) | 2,179 (33.1%) |  |
| Poor | 807 (9.3%) | 254 (11.9%) | 553 (8.4%) |  |
| Memory |  |  |  | <0.001 |
| Good | 1,967 (22.6%) | 405 (19.0%) | 1,562 (23.7%) |  |
| Fair | 4,377 (50.2%) | 1,011 (47.4%) | 3,366 (51.1%) |  |
| Poor | 2,372 (27.2%) | 719 (33.7%) | 1,653 (25.1%) |  |
| Function limitation | 11.5 (3.8) | 12.4 (4.6) | 11.2 (3.5) | <0.001 |
| Depressive symptoms |  |  |  | <0.001 |
| Yes | 2,055 (23.6%) | 668 (31.3%) | 1,387 (21.1%) |  |
| No | 6,661 (76.4%) | 1,467 (68.7%) | 5,194 (78.9%) |  |
| Cognitive function | 14.0 (10.0-18.0) | 13.0 (9.0-18.0) | 15.0 (10.0-19.0) | <0.001 |
| SPPB |  |  |  | <0.001 |
| Good | 7,165 (82.2%) | 1,607 (75.3%) | 5,558 (84.5%) |  |
| Fair | 1,143 (13.1%) | 363 (17.0%) | 780 (11.9%) |  |
| Poor | 408 (4.7%) | 165 (7.7%) | 243 (3.7%) |  |
| Height | 159.0 (8.1) | 157.5 (8.2) | 159.4 (8.0) | <0.001 |
| Weight | 59.5 (10.6) | 58.0 (10.7) | 59.9 (10.6) | <0.001 |
| Total Cholesterol | 191.6 (33.5) | 194.8 (34.8) | 190.6 (32.9) | <0.001 |
| Triglycerides | 107.1 (84.1-142.0) | 110.5 (86.7-144.5) | 106.4 (83.8-141.6) | 0.014 |
| High Density Lipoprotein Cholesterol | 50.8 (13.3) | 51.7 (14.2) | 50.6 (13.0) | 0.001 |
| C-Reactive Protein | 1.3 (0.7-2.1) | 1.4 (0.7-2.4) | 1.2 (0.7-2.1) | 0.003 |
| **Relationship level** |  |  |  |  |
| Marital status |  |  |  | <0.001 |
| Married | 7,250 (83.2%) | 1,672 (78.3%) | 5,578 (84.8%) |  |
| No | 1,466 (16.8%) | 463 (21.7%) | 1,003 (15.2%) |  |
| **Community level** |  |  |  |  |
| Toilet |  |  |  | 0.028 |
| Yes | 6,411 (73.6%) | 1,531 (71.7%) | 4,880 (74.2%) |  |
| No | 2,305 (26.4%) | 604 (28.3%) | 1,701 (25.8%) |  |
| Main terrain |  |  |  | <0.001 |
| Plain | 3,929 (45.1%) | 869 (40.7%) | 3,060 (46.5%) |  |
| Hill | 2,589 (29.7%) | 685 (32.1%) | 1,904 (28.9%) |  |
| Mountainous region | 1,604 (18.4%) | 423 (19.8%) | 1,181 (17.9%) |  |
| Plateau | 355 (4.1%) | 77 (3.6%) | 278 (4.2%) |  |
| Basin | 239 (2.7%) | 81 (3.8%) | 158 (2.4%) |  |
| Rainy days in the past year | 40.0 (20.0-64.0) | 40.0 (22.0-70.0) | 38.0 (20.0-60.0) | <0.001 |
| Distance to the bus stop | 0.1 (0.0-2.0) | 0.3 (0.0-2.0) | 0.1 (0.0-2.0) | 0.705 |
| Electricity supply | 23.6 (1.5) | 23.5 (1.8) | 23.6 (1.4) | 0.002 |
| Degree of crowdness | 5.0 (4.0-6.0) | 5.0 (4.0-6.0) | 5.0 (4.0-6.0) | 0.039 |

Note: SPPB, Short Physical Performance Battery.

**Appendix Table S9 AUC-ROC Comparison of Different ML Models on the Test Set of Pain and Non-pain Populations**

| **ML models** | **LR** | **NB** | **RF** | **XGBoost** | **ANN** |
| --- | --- | --- | --- | --- | --- |
| **Pain** |  |  |  |  |  |
| LR | \ | 0.021 | 0.996 | 0.540 | 0.464 |
| NB | 0.021 | \ | 0.022 | 0.129 | 0.083 |
| RF | 0.996 | 0.022 | \ | 0.539 | 0.669 |
| XGBoost | 0.540 | 0.129 | 0.539 | \ | 0.866 |
| ANN | 0.464 | 0.083 | 0.669 | 0.866 | \ |
| **Non-pain** |  |  |  |  |  |
| LR | \ | 0.111 | 0.796 | ＜0.001 | 0.111 |
| NB | 0.111 | \ | 0.122 | ＜0.001 | 0.259 |
| RF | 0.796 | 0.122 | \ | ＜0.001 | 0.446 |
| XGBoost | ＜0.001 | 0.002 | ＜0.001 | \ | ＜0.001 |
| ANN | 0.111 | 0.259 | 0.446 | ＜0.001 | \ |

Note: LR, logistic regression; NB, naive bayesian; RF, random forest; XGBoost, extreme gradient boosting; ANN, artificial neural network; AUC-ROC, area under the receiver operating characteristic curve.

**Appendix Table S10 Performance of the LR Model for Predicting Falls Stratified by Age**

| **Age group** | **Threshold** | **AUC-ROC (95%CI)** | **Accuracy** | **Sensitivity** | **Specificity** | **Brier score** |
| --- | --- | --- | --- | --- | --- | --- |
| 45-60 |  |  |  |  |  |  |
| Pain | 0.295 | 0.720  (0.683-0.759) | 0.718 | 0.734 | 0.633 | 0.189 |
| Non-pain | 0.167 | 0.677  (0.631-0.731) | 0.812 | 0.813 | 0.786 | 0.145 |
| ≥60 |  |  |  |  |  |  |
| Pain | 0.514 | 0.722  (0.686-0.766) | 0.690 | 0.704 | 0.660 | 0.206 |
| Non-pain | 0.341 | 0.676  (0.634-0.715) | 0.731 | 0.750 | 0.612 | 0.190 |

**Appendix Figure S1 Partial Dependence Plots for Key Predictors of Fall Risk in Middle-aged and Older Adults with Pain**

**
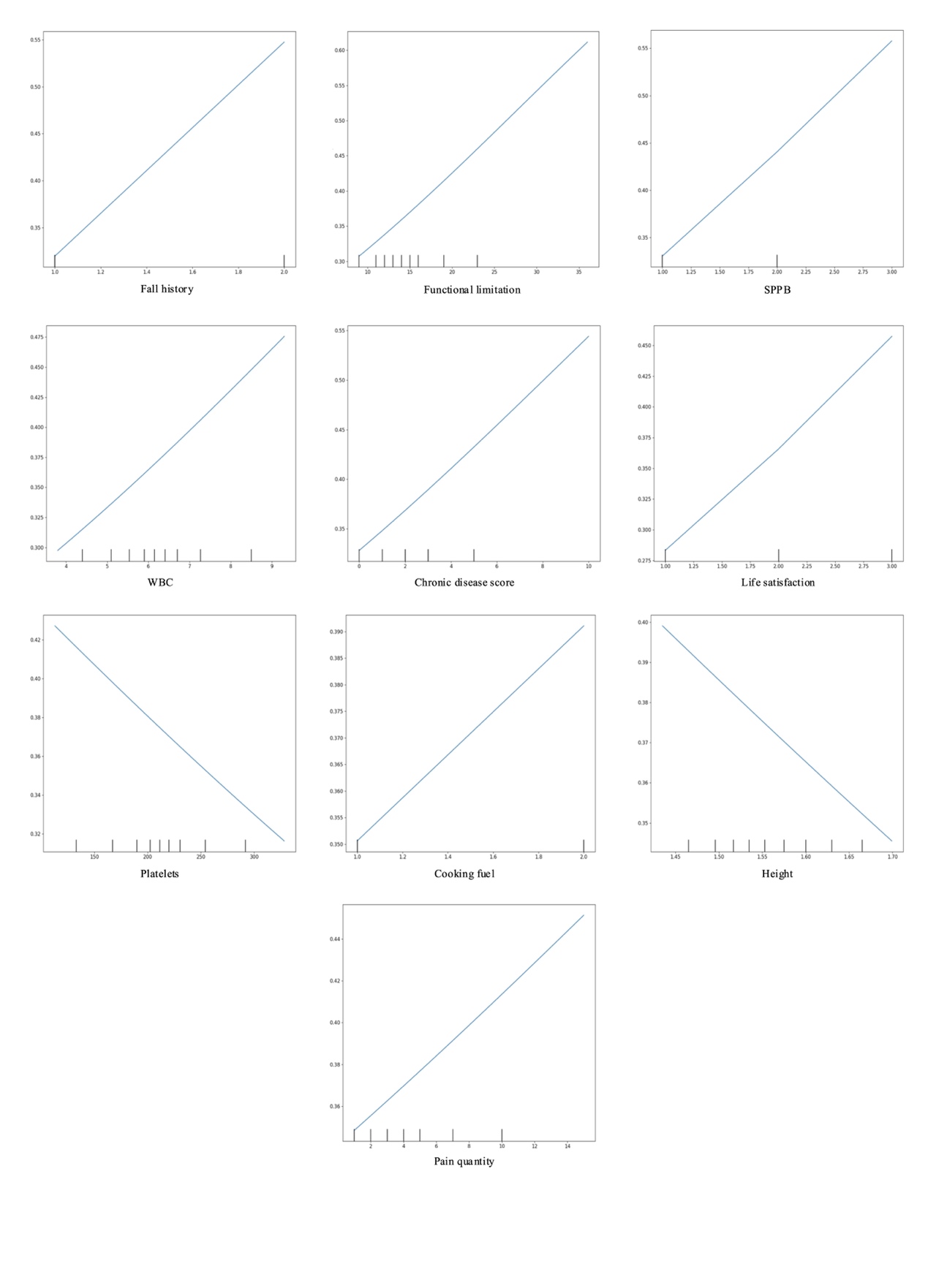
**

Note: Fall history: 1 = No, 2 = Yes; SPPB: 1=Good, 2=Fair, 3=Poor; Life satisfaction: 1=Good, 2=Fair, 3=Poor; Cooking fuel: 1=non-pollution, 2=pollution.

Abbreviations: SPPB, Short Physical Performance Battery; WBC, White Blood Cell; HbA1c, Glycated Hemoglobin

**Appendix Figure S2 Feature Importance Ranking (Top 10) with SHAP Summary Plots for Four Alternative Machine Learning Models among Pain Population**


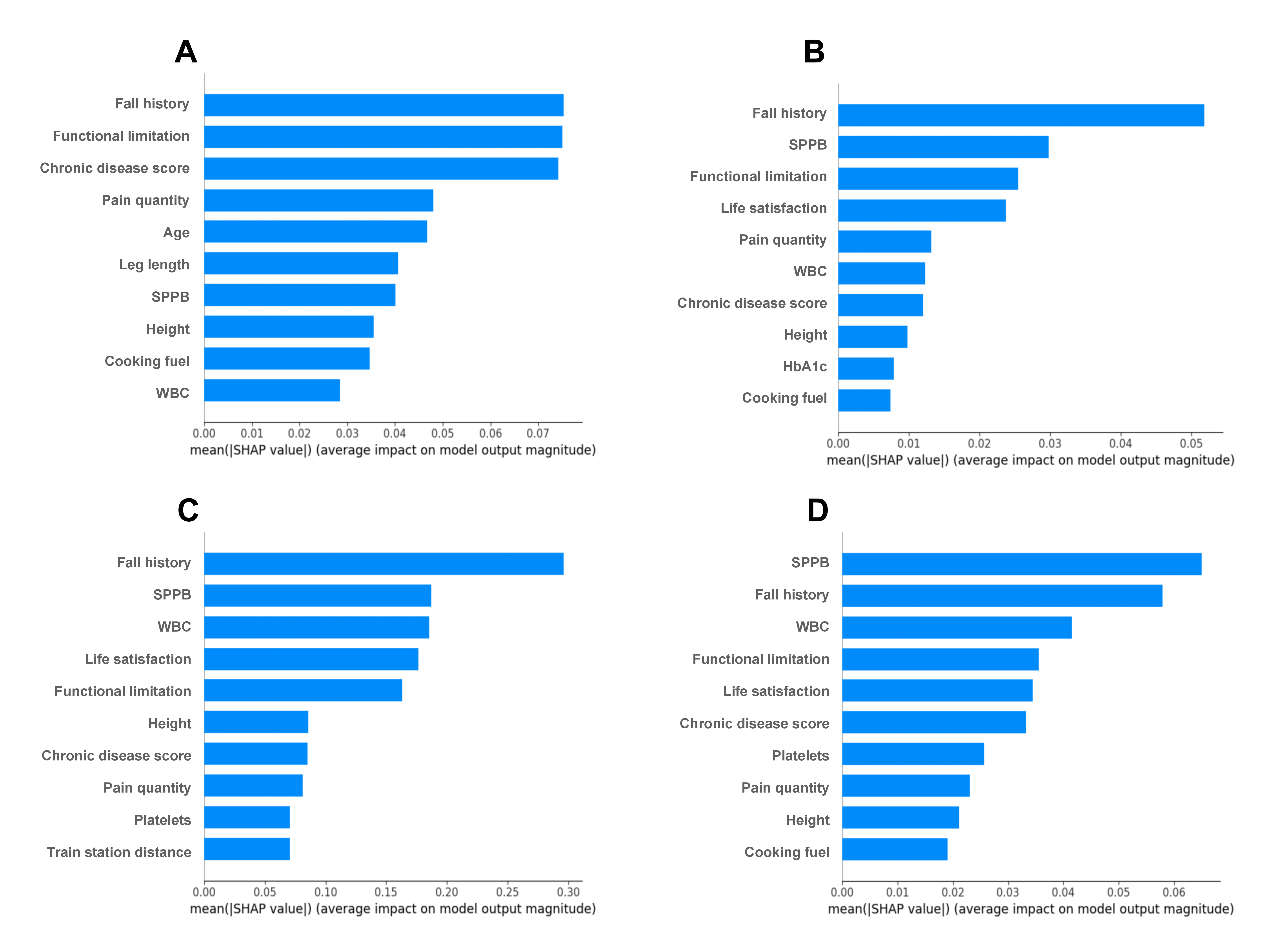


Note: (A) NB model, (B) RF model, (C) XGBoost model, (D) ANN model.

Abbreviations: SPPB, Short Physical Performance Battery; WBC, White Blood Cell; HbA1c, Glycated Hemoglobin

**
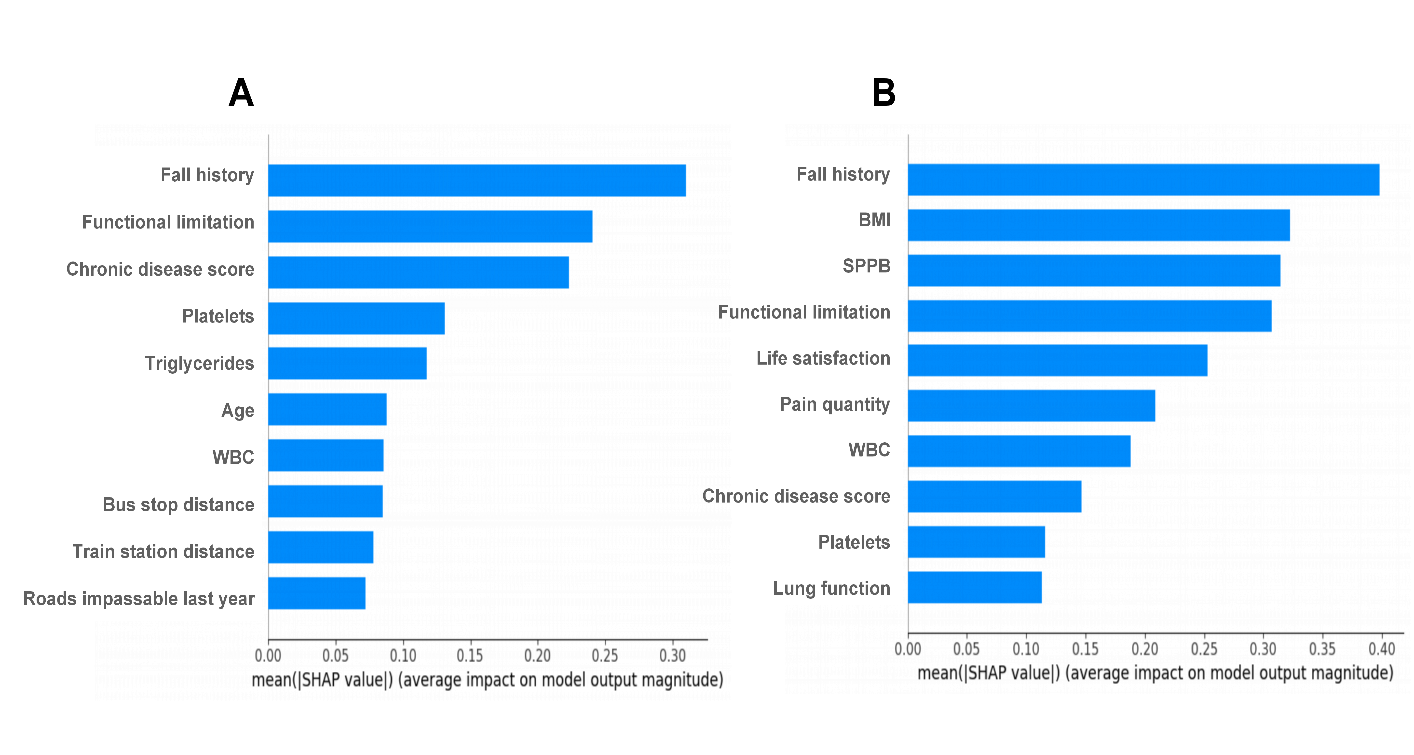
****Appendix Figure S3 Importance Ranking (Top 10) with the SHAP Summary Plot for the Logistic Regression Models Stratified by Age among Pain Population**

Note: (A) Age 45-60 model, (B) Age 60+ model.

Abbreviations: WBC, White Blood Cell; BMI, Body Mass Index; SPPB, Short Physical Performance Battery
